# Danshensu inhibits the IL-1β-induced inflammatory response in chondrocytes and osteoarthritis possibly via suppressing NF-κB signaling pathway

**DOI:** 10.1186/s10020-021-00329-9

**Published:** 2021-07-20

**Authors:** Zhixian Xu, Tie Ke, Yongfa Zhang, Licheng Guo, Feng Chen, Wubing He

**Affiliations:** 1 Shengli Clinical Medical College of Fujian Medical University, Fuzhou, 350004 Fujian Province People’s Republic of China; 2 Department of Emergency Surgery, Fujian Provincial Hospital, No.134 East Street, Gulou District, Fuzhou, 350001 Fujian Province People’s Republic of China; 3 Fujian Provincial Key Laboratory of Emergency Medicine, Fujian Provincial Hospital, Fuzhou, 350001 Fujian People’s Republic of China

**Keywords:** Osteoarthritis, Danshensu, IL-1β, Inflammatory response, NF-κB

## Abstract

**Purpose:**

Osteoarthritis (OA) is the most common inflammatory disease associated with pain and cartilage destruction. Interleukin (IL)-1β is widely used to induce inflammatory response in OA models. This study aimed to explore the role of Danshensu (DSS) in IL-1β-induced inflammatory responses in OA.

**Methods:**

IL-1β was used to induce chondrocyte inflammation. Cell viability was evaluated by Cell Counting Kit-8 (CCK-8) assay. IL-6, COX-2, TNF-α, and iNOS mRNA levels were detected by qRT-PCR. MMP3, MMP13, ADAMTS4, ADAMTS5, Aggrecan, Collagen, p-IκBα, and p-p65 protein levels were detected by Western blot. An OA mouse model was established by surgical destabilization of the medial meniscus (DMM), and the Osteoarthritis Research Society International (OARSI) score was evaluated by H&E staining.

**Results:**

DSS did not affect the levels of inflammatory indicators including IL-6, COX-2, TNF-α, iNOS, PEG2, and NO but suppressed COX-2 and iNOS protein expression in IL-1β treated chondrocytes. In addition, DSS downregulated IL-1β-enhanced expression of MMP3, MMP13, ADAMTS4, and ADAMTS5 and upregulated aggrecan and collagen expression. Moreover, DSS significantly inhibited IL-1β-induced phosphorylation of p-IκBα and p-p65 in a dose-dependent manner in chondrocytes, suggesting it plays a role in the NF-κB signaling pathway. Furthermore, DSS significantly reduced DMM-induced cartilage OARSI score in mice, further demonstrating its protective role in OA progression in vivo.

**Conclusions:**

Our study revealed the protective role of DSS in OA, suggesting that DSS might act as a potential treatment for OA.

## Introduction

Osteoarthritis (OA) has become a common joint disease, which is characterized by pain and could lead to disability in the adult population, affecting approximately 240 million people worldwide (Rim et al. 2020; Nelson 2018). Increasing evidence indicates that approximately 30% of OA patients may undergo neuropathic pain (Schomberg et al. 2012). OA is a disease with cartilage degeneration, chondrocyte inflammation, and subchondral sclerosis of the whole joint, of which cartilage degeneration and inflammation response play essential roles in OA progression (Goldring and Goldring 2016). Low-level inflammation can contribute to the degenerative changes and development of peripheral sensitization and nociceptive pain (Krustev et al. 2015; McDougall et al. 2009). Many therapeutic strategies for OA mainly aimed to alleviate inflammatory pain, reduce stiffness, maintain cartilage functional capacities, and improve patients’ quality of life (Felson 2006; Conaghan et al. 2019). However, identifying effective treatment strategies in this common chronic OA, specifically OA pain, is still a challenge.

Aggrecan and collagen are the main components of the extracellular matrix (ECM). Downregulation of their expression leads to cartilage degradation (Pattoli et al. 2005). Disintegrin and metalloproteinase with thrombospondin motifs (ADAMTS) enzymes, especially ADAMTS-4 and ADAMTS-5, are considered the primary aggrecanases with the ability to cleave aggrecans (Stanton 2005). In addition, IL-1β can induce cartilage degradation by promoting the expression of matrix metalloproteinases (MMPs) in chondrocytes (Yin and Lei 2018; Zhang 2019). Previous studies have shown that inflammatory factors like IL-1β play a crucial role in OA progression by regulating nuclear factor-kappa B (NF-κB) (Jotanovic et al. 2012; Berenbaum 2004). NF‐κB is a dimer composed of two proteins, p65 and IκBα, at the resting state. IκBα phosphorylation and subsequent degradation lead to p65 phosphorylation, activation, and translocation from the cytoplasm to the nucleus, where it promotes the transcription of its target genes (Mendes et al. 2002), including many inflammation-related genes such as MMPs, iNOS, and IL-6 (Mitchell and Carmody 2018). NF-κB signaling pathway has been well identified to participate in the inflammatory progress and considered a potential target in OA treatment (Saklatvala 2007).

In the last decades, replacement surgery has been the primary approach for OA treatment to control the symptoms (Brosseau 2003). Based on the identification that inflammation is responsible for OA progression, specifically OA pain, anti-inflammation substances are potentially applied for treating OA to inhibit chondrocyte inflammation. For instance, trans-cinnamaldehyde (TCA) inhibits IL-1β-stimulated inflammation in chondrocytes by suppressing NF-κB and p38-JNK pathways and exhibits chondrocyte protective effects in a rat OA model (Xia 2019). Schisandrin B ameliorates chondrocyte inflammation and OA progression through inhibiting NF-κB activation and MAPK signaling pathway (Ran 2018). In addition, nonsteroidal anti-inflammatory drugs (NSAIDs) are widely applied for OA treatment to reduce chronic pain (Argoff 2011). Despite these advantages, it is necessary to identify safer and more effective therapeutic agents for OA.

Danshensu (DSS) is a bioactive agent isolated from an edible traditional Chinese medicine salvia miltiorrhiza (dan shen) (Cao 2019). DSS has been reported to have an anti-inflammatory effect involving TLR2 (Toll-like receptor 2) and macrophages through the NF-κB signaling pathway (Ye 2020). In addition, DSS can also significantly ameliorate colon inflammation in dextran sulfate sodium-induced colitis (Wen 2013). In chronic kidney disease, the combination of rhein (RH), a natural chondroprotective agent, and DSS shows a better protective effect through improving renal function, blood supply, fibrotic degree, and suppressing pro-inflammatory cytokines via deactivating NK-κB signaling pathway (Guan 2015). However, the roles of DSS in OA and its underlying specific mechanism remain unclear.

Here, we demonstrated that DSS downregulated IL-1β-induced over-production of inflammatory cytokines including nitric oxide (NO), tumor necrosis factor (TNF)-α, prostaglandin E_2_ (PGE2), and IL-6, as well as the expression of cyclooxygenase-2 (COX-2), inducible NO synthase (iNOS), MMP3, MMP13, ADAMTS4, and ADAMTS5 in chondrocytes. Meanwhile, DSS promoted IL-1β-induced defective expression of aggrecan and collagen in chondrocytes. Specifically, our results indicated that DSS inhibited IL-1β-activated NF-κB signaling pathway in chondrocytes. Overall, our study indicated that DSS could efficiently prevent OA progression in vivo and provided a potential treatment strategy for OA treatment.

## Materials and methods

### Animal experiments

A total of sixty 10–12-week-old C57BL/6 male mice from the Animal Center of Chinese Academy of Sciences Shanghai, China, were used in the study. The OA mouse model was established using surgical destabilization of the medial meniscus (DMM), as previously described (Liu 2020). These mice were randomly divided into three groups, namely the sham group, DMM group, and DMM + DSS group, with 10 mice per group. The mice in the DSS group were then intraperitoneally injected with 40 mg/kg DSS, and mice in the Sham and DMM group were injected with PBS as the control. The mice were sacrificed by injecting overdose pentobarbital sodium at the end of the 8th week. The animal experiment was performed according to the National Institutes of Health Guide for Care and Use of Laboratory Animals and approved by Fujian Provincial Hospital.

### Chondrocyte isolation and culture

Chondrocytes were isolated from C57BL/6 male mice as previously described (Xu 2020). The isolated chondrocytes were cultured in low-glucose DMEM containing 10% FBS, 100 U/mL penicillin, and 100 mg/mL streptomycin. Danshensu (purity 99%) was purchased from the National Institute for the Control of Pharmaceutical and Biological Products (Beijing, China) and dissolved in DMSO to obtain the desired concentrations. When needed, cells were treated with 10 ng/ml IL-1β and different concentrations of DSS for different times as indicated and collected for subsequent experiments.

### Cell viability

Chondrocytes were plated into 96-well plates at a density of ~ 5000 cells/well. Cells were treated with different concentrations of DSS (0, 1.25, 2.5, 5, 10, 20, and 40 μM) for 24 h or 48 h, respectively, collected, and subjected to cell viability assay using Cell Counting Kit-8 (CCK-8) solution (Dojindo Molecular Technologies, Inc.). The absorbance at 450 nm was detected as an indicator of the cell viability using a microplate reader (Bio-Rad, Hercules, CA, USA).

### qRT-PCR 

Total RNA of chondrocytes was extracted with TRIzol reagent according to the manufacturer's instructions. Approximately 1 μg RNA was transcribed to cDNA using Double-Strand cDNA Synthesis Kits, and qRT-PCR was performed using the SYBR-based CFX96 Real-Time PCR System. The relative expression change of targets was analyzed using the 2^−ΔΔCt^ method (Schmittgen and Livak 2008), with β-actin as the internal reference. The primers used in this study were TNF-α forward 5′-CAGGCGGTGCCTATGTCTC-3′ and reverse 5′-CGATCACCCCGAAGTTCAGTAG- 3′, IL-6 forward 5'-TACCACTTCA CAAGTCGGAGGC-3′ and reverse 5′-CTGCAAGTGCATCATCGTTGTTC-3', iNOS forward 5′-CTCTTCGACGACCCAGAAAAC-3' and reverse 5′-CAAGGCCATGAA GTGAGGCTT- 3′, Cox-2 forward5′-CACCCTGACATAGACAGTGAAAG-3′ and reverse 5′-CTGGGTCACGTTGGATGAGG-3′, as well as β-actin forward 5′-AGCCA TGTACGTAGCCAT CC-3′ and reverse 5′-CTCTCAGCAGTGGTGGTGAA-3′.

### Western blot

The total proteins from chondrocytes were extracted using RIPA lysis buffer, and protein concentration was determined with the BCA protein assay kit according to the manufacturer’s instruction. Approximately 40 μg protein samples were separated by 12% SDS-PAGE and transferred onto PVDF membranes. After blocking with 5% nonfat milk, the membranes were induced with primary antibodies against COX-2 (1:1000, Abcam), iNOS (1:1000, Abcam), MMP-3 (1:1000, Abcam), MMP-13 (1:1000, Abcam), ADAMTS-4 (1:1000, Abcam), ADAMTS-5 (1:1000, Abcam), aggrecan (1:1000, Abcam), collagen II (1:1000, Abcam), p65 (1:2000, Abcam), p-p65 (1:2000, Abcam), IkBα (1:2000, Abcam), p-IkBα (1:2000, Abcam), lamin B (1:1000, Abcam) and β-actin (1:5000, Abcam) overnight at 4 °C. Then the membranes were washed and incubated with HRP-conjugated secondary antibodies (1:3000) for 2 h. Finally, signals on the membranes were detected using the Enhanced Chemiluminescence (ECL) kit and quantified by the Quantity ONE software (Bio-Rad, USA).

### Histological analysis

Knee joints were isolated from mice and fixed with 4% formaldehyde. Calcium in the knee joints was removed using a decalcifying solution for 21 days. At last, the knee joints were embedded in paraffin and coronally sectioned. The sections were stained with Safranin O or hematoxylin & eosin (H&E) as previously described (Jia 2020) and scored using the Osteoarthritis Research Society International (OARSI) scoring system to determine the extent of cartilage deterioration as previously reported (Pritzker 2006). Grade 0 was for intact surface and cartilage; Grade 1 for intact surface only; Grade 2 for surface discontinuity, Grade 3 for vertical fissures; Grade 4 for erosion, Grade 5 for denudation, and Grade 6 for deformation.

### Immunofluorescence assay

Chondrocytes that had been pretreated with 10 μM DSS for 1 h and then treated with 10 ng/mL IL-1β for 30 min were plated on glass coverslips. After fixation in cold methanol, cells were incubated with primary antibody against collagen II (1:500) overnight at 4 °C, and then with fluorescein isothiocyanate-conjugated secondary antibody for 1 h and DAPI solution for 5 min. After that, cells were observed and photographed using a Leica fluorescence microscope.

### Data analysis

All experiments were performed at least three independent times. Data were presented as mean ± standard deviation (SD). The difference between two groups was analyzed by Student’s t-tests and among multiple groups were analyzed with one-way ANOVA, with p < 0.05 as the significant threshold.

## Results

### Low-dose DSS showed no cytotoxicity to osteoarthritic chondrocytes

**Fig. 1:**
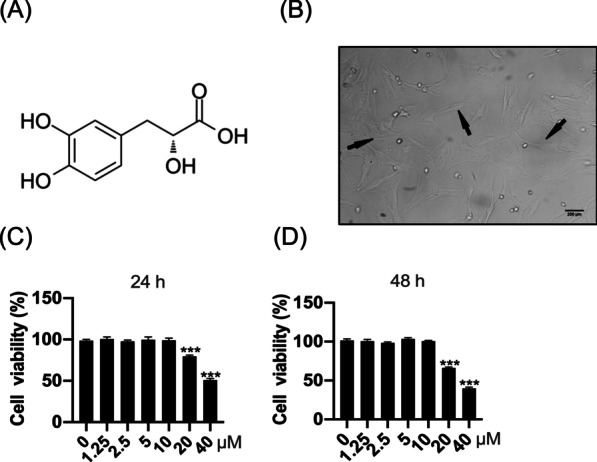
The cytotoxicity of DSS on chondrocytes. **A** The structure of DSS. **B** The morphology of primary chondrocytes. Scale bar = 200 μm. **C**, **D** Chondrocyte viability was detected by the CCK-8 kit after treatment with different concentrations of DSS for 24 h (**C**) and 48 h (**D**), respectively. ***p < 0.001

The chemical structure of DSS was shown in Fig. 1A. To explore its role in OA progression, we first isolated chondrocytes from C57BL/6 male mice. The morphology of the isolated primary chondrocytes was evaluated and shown in Fig. 1B, which indicated that chondrocytes were successfully isolated and could be applied for subsequent experiments. We then treated the chondrocytes with DSS at different concentrations (0, 1.25, 2.5, 5, 10, 20, and 40 μM) for 24 and 48 h, respectively. The CCK-8 assay indicated that chondrocyte viability was significantly reduced after treatment with 20 and 40 μM DSS for 24 h (p < 0.001) (Fig. 1C) and 48 h (p < 0.001) (Fig. 1D), respectively, but not after treatment with < 10 μM DSS, indicating that DSS at low concentration (≤ 10 μM) exhibited no obvious cytotoxicity to chondrocytes. Hence, we selected DSS at 2.5, 5, and 10 μM to explore its role in OA progression.

### DSS inhibited IL-1β-induced chondrocyte inflammation

**Fig. 2:**
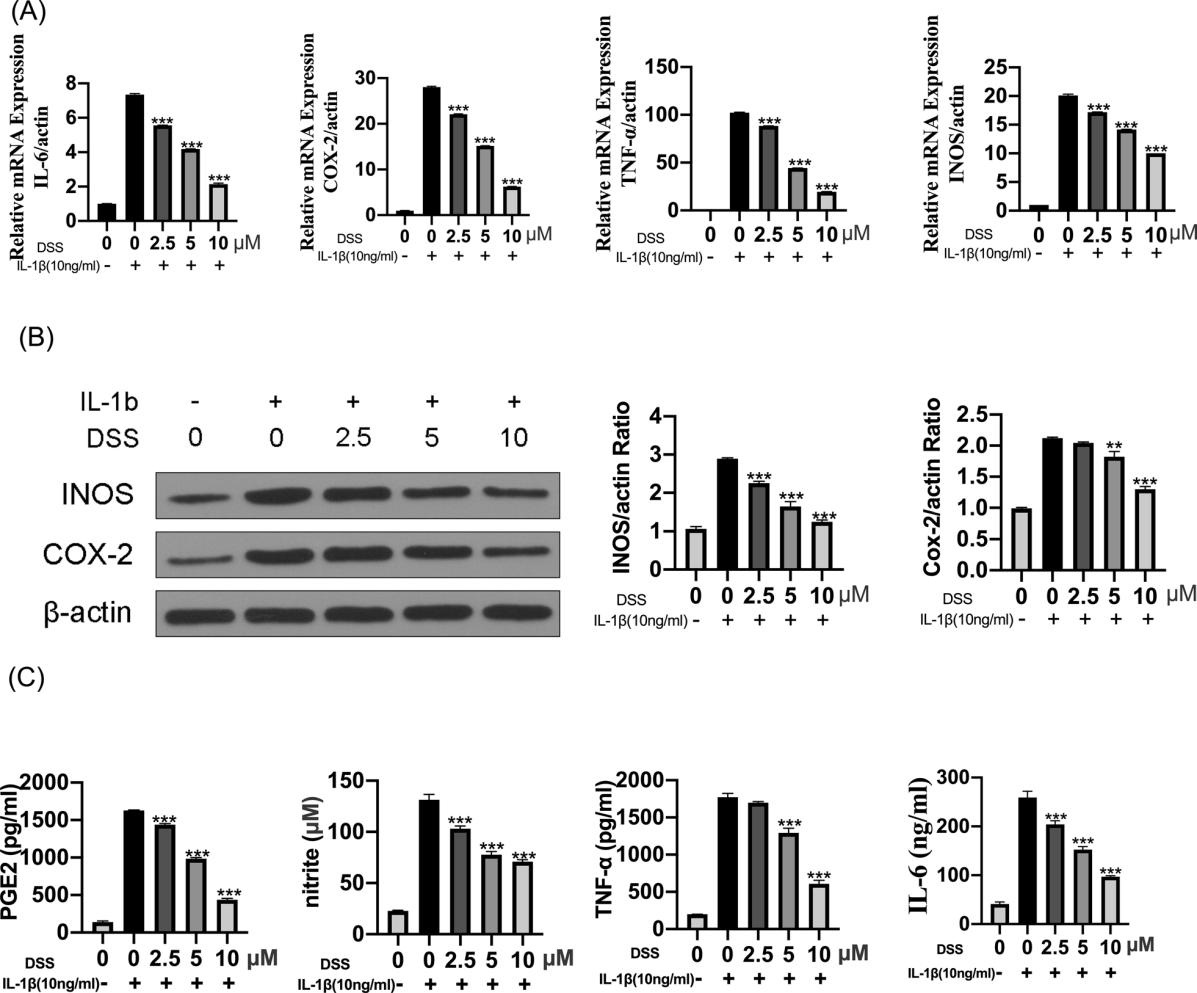
DSS inhibited IL-1β-induced chondrocytes inflammation. The chondrocytes were pretreated with 2.5, 5, and 10 μM DSS for 24 h and stimulated with 10 ng/ml IL-1β for 2 h. **A** The mRNA levels of inflammation-related markers IL-6, COX-2, TNF-α, and iNOS were evaluated by qRT-PCR. **B** The protein levels of iNOS and COX-2 was detected by Western blot with β-actin as the internal reference. **C** The production of PGE2, TNF-α, NO, and IL-6 were detected by ELISA. **p < 0.01; ***p < 0.001

To investigate the effects of DSS in IL-1β-induced chondrocyte inflammation, chondrocytes were pretreated with DSS for 24 h at 2.5, 5, and 10 μM, respectively, and treated with 10 ng/ml IL-1β for 2 h. qRT-PCR analyses showed that DSS treatment attenuated IL-1β-induced expression of IL-6, COX-2, TNF-α, and iNOS in a dose-dependent manner (all p < 0.001) (Fig. 2A). Similarly, Western blot results also confirmed the dose-dependent downregulatory effects of DSS on IL-1β-elevated protein levels of inflammation-related indicators, including COX-2 and iNOS (p < 0.01) (Fig. 2B). Further, the production of NO, PGE2, IL-6, and TNF-α in the culture supernatant was detected by ELISA kit, and the results showed that DSS treatment significantly inhibited IL-1β-induced secretion of NO, PGE2, IL-6, and TNF-α (all p < 0.001) (Fig. 2C). These results indicated that DSS inhibited IL-1β-induced chondrocyte inflammation.

### DSS protected chondrocytes against IL-1β-caused ECM degradation

**Fig. 3:**
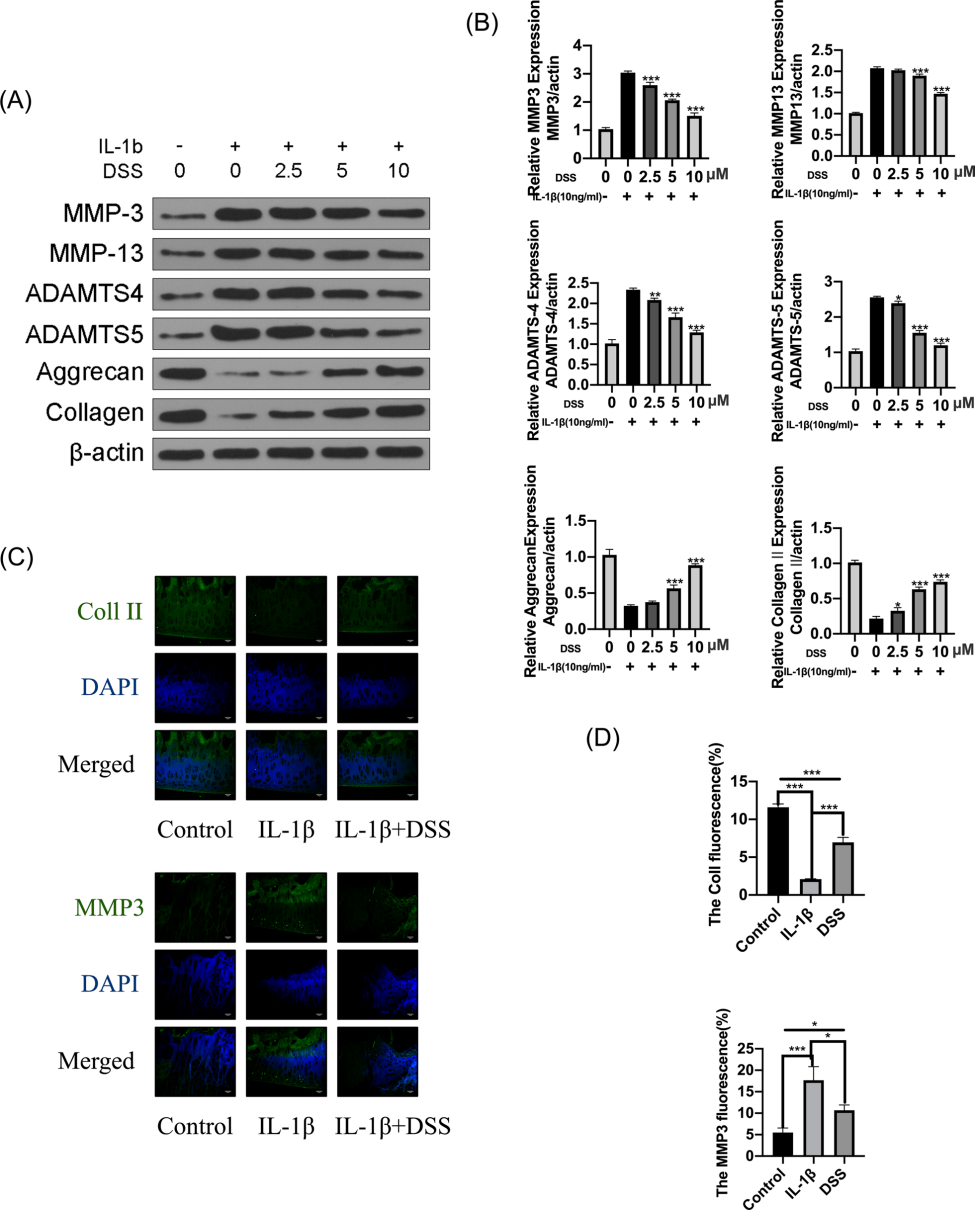
DSS protected chondrocytes against IL-1β-induced ECM degradation. The chondrocytes were treated with 2.5, 5, and 10 μM DSS for 24 h and then stimulated by 10 ng/ml IL-1β for 2 h. **A**, **B** Protein levels of MMP3, MMP13, ADAMTS4, ADAMTS5, aggrecan, and collagen II in chondrocytes (**A**), and their quantification by the Quantity ONE software (**B**). **C**, **D** Representative fluorescence images of collagen II and MMP3 detected by immunofluorescent staining assay (**C**) and the percentage of cell immunofluorescence in chondrocytes that were pretreated with 10 μM DSS for 24 h and stimulated by 10 ng/ml IL-1β for 2 h (**D**). Scale bar = 50 μm. *p < 0.05, **p < 0.01, and ***p < 0.001

To explore the role of DSS pretreatment on ECM degradation, we detected the protein level of ECM-related genes in IL-1β-treated chondrocytes by Western blot. We found that IL-1β treatment decreased aggrecan and collagen II expressions while significantly promoted ADAMTS-4, ADAMTS-5, MMP-3, and MMP-13 expressions. However, these IL-1β-induced changes were significantly reversed by DSS pretreatment, meaning that DSS pretreatment increased aggrecan and collagen II expression and reduced ADAMTS-4, ADAMTS-5, MMP-3, and MMP-13 expression (p < 0.05) (Fig. 3A, B). In addition, immunofluorescence assay indicated that DSS significantly rescued IL-1β-stimulated collagen II degradation (p < 0.001) and inhibited IL-1β-induced MMP3 overexpression in chondrocytes (p < 0.05) (Fig. 3C, D). These results indicated that DSS efficiently protected chondrocytes against IL-1β-induced ECM degradation.

### DSS inhibited IL-1β-induced activation of NF-κB pathway in chondrocytes

**Fig. 4:**
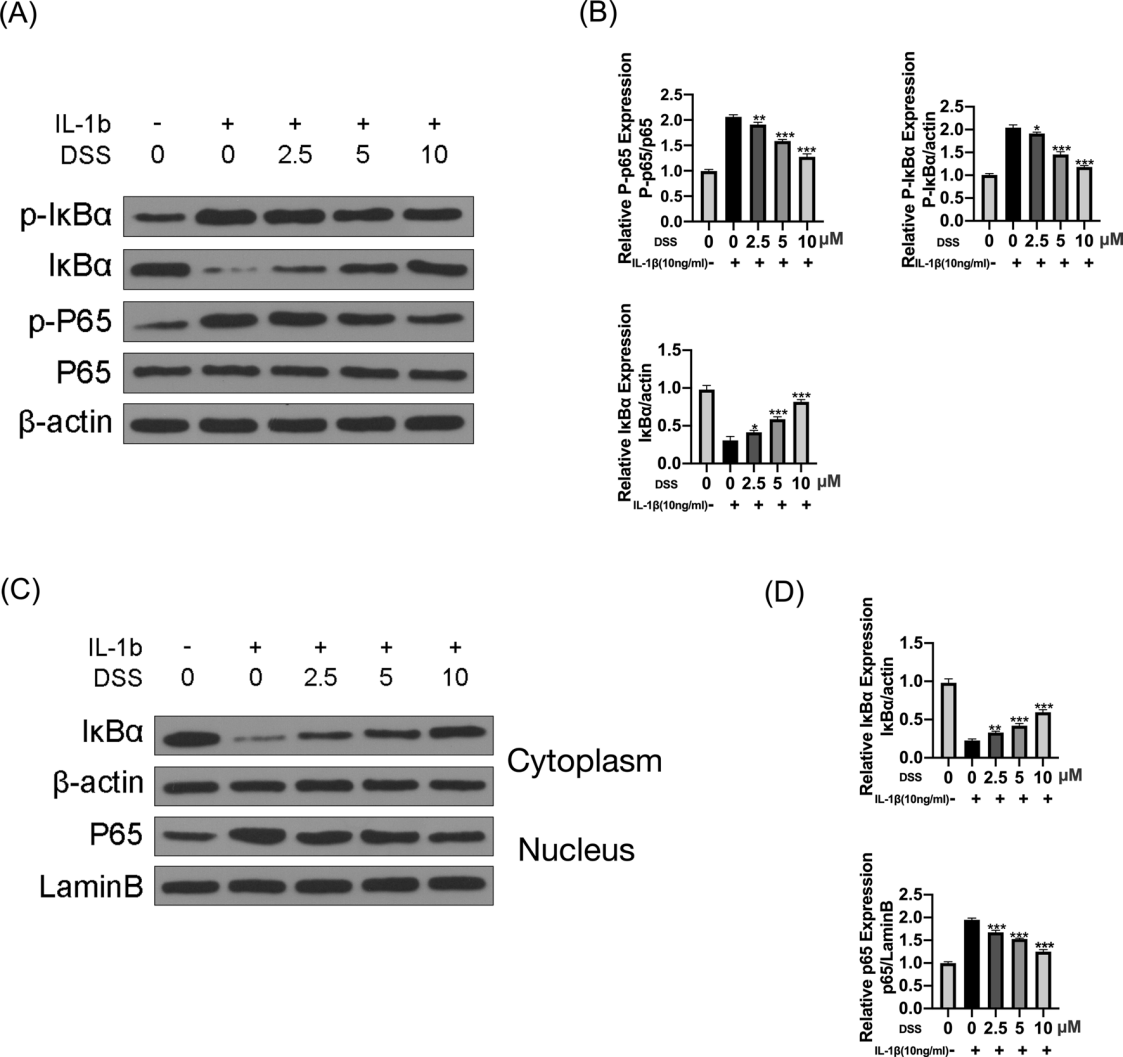
DSS inhibited IL-1β-induced activation of the NF-κB pathway in chondrocytes. The chondrocytes were treated 2.5, 5 and 10 μM DSS for 24 h and then induced with or without 10 ng/ml IL-1β for 2 h. The protein levels of p65, p-p65, IκBα, and p-IκBα were detected by Western blot (**A**) and quantitative analysis (**B**). The protein levels of IκBα in the cytoplasm and p65 in the nucleus of chondrocytes were detected by Western blot (**C**) and quantitative analysis (**D**). *p < 0.05, **p < 0.01, and ***p < 0.001

We next evaluated the effects of DSS on the NF-κB pathway. The results indicated that IL-1β significantly increased p-p65 and p-IκBα levels in chondrocytes, leading to IκBα degradation. Meanwhile, DSS dramatically inhibited IL-1β-elevated p-IκBα and p-p65 levels in a dose-dependent manner (p < 0.05) (Fig. 4A, B). For further confirmation, we examined IκBα level in the cytoplasm and p65 in the nucleus of IL-1β-treated chondrocytes by Western blot. The results indicated that IL-1β treatment reduced IκBα level in the cytoplasm and promoted p65 translocating to the nucleus. These effects were significantly reversed by DSS pretreatment in a dose-dependent manner (p < 0.01) (Fig. 4C, D), indicating that DSS pretreatment efficiently inhibited IL-1β-induced activation of the NF-κB pathway in OA chondrocytes.

### DSS attenuated OA progression in the OA mouse model

**Fig. 5:**
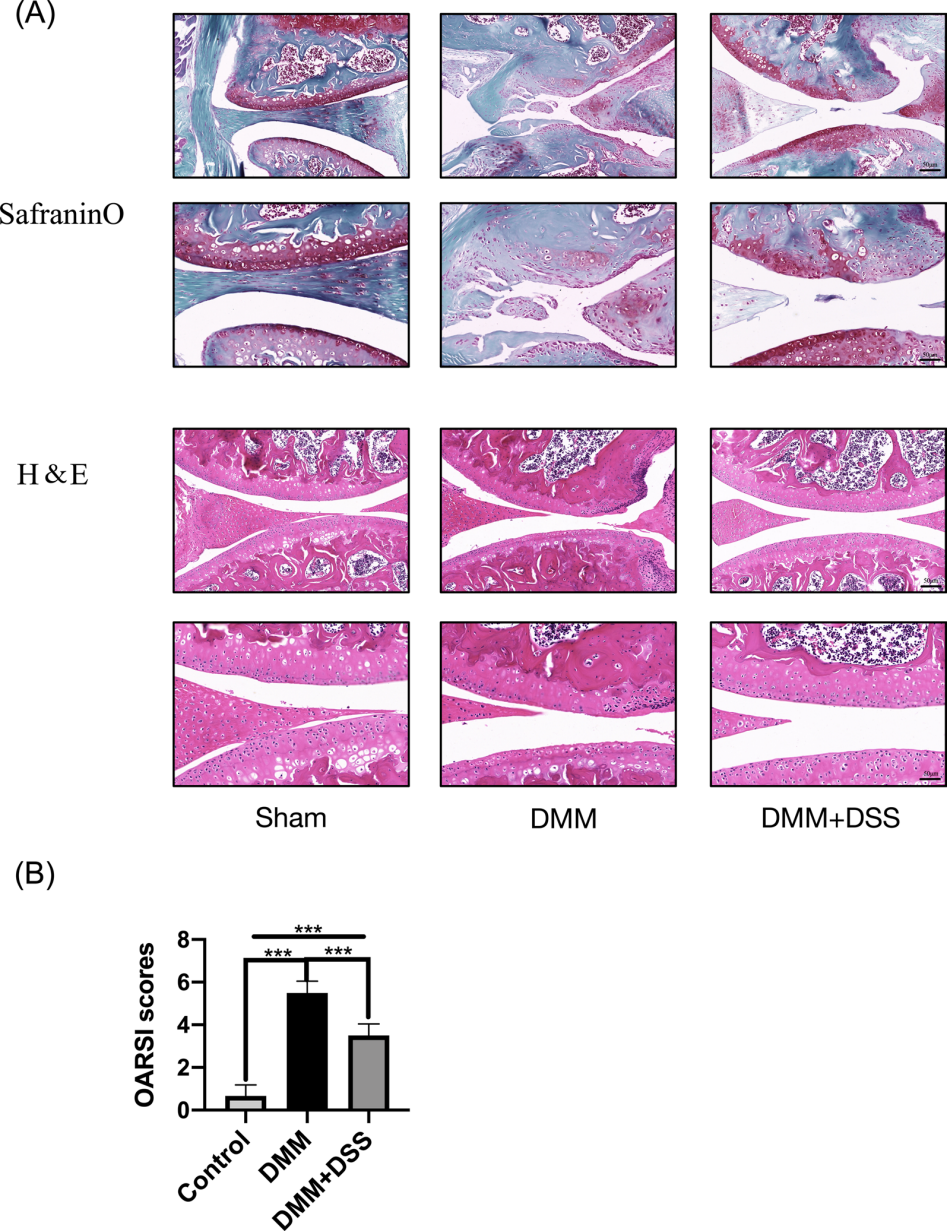
DSS attenuated OA progression in mice model. **A** Representative images of safranin O and H&E staining of cartilage sections from different groups. **B** The cartilage OARSI scores of different groups. Scale bar = 50 μm. Ten mice in each group, ***p < 0.001

To confirm whether DSS played a protective role in OA progression in vivo, the surgical mouse OA model was established, and DSS was applied. The results showed that mice in the sham group had smooth and intact cartilage surface while mice in the DMM group had destructed cartilage surface, eroded cartilage, and apparent hypocellularity. Moreover, DSS suppressed cartilage degradation observed in the DMM group (Fig. 5A). Consistent with the observed phenotypes in the cartilage of mice, DSS significantly reduced cartilage OARSI score elevated by DMM (p < 0.001) (Fig. 5B). These data demonstrated that DSS efficiently attenuated OA progression in vivo.

## Discussion

Danshensu, a herbal preparation used in traditional Chinese medicine, has been reported to exhibit protective effects in various human diseases, including Parkinson's disease (Han 2019) and Lewis lung carcinoma xenografts in mice (Cao 2019). In addition, DSS has anti-tumor activity in B16F10 melanoma by inhibiting angiogenesis and tumor cell invasion (Zhang 2010). Recently, DSS has been reported to have anti-immune activity (Ye 2020; Guan 2015), which attracted us to focus on its role in OA progression. Interestingly, we found that DSS treatment significantly decreased IL-1β-enhanced expression of IL-6, COX-2, TNF-α, and iNOS in a dose-dependent manner. Meanwhile, DSS decreased IL-1β-induced expression of inflammatory makers iNOS and COX-2 at the protein level. Moreover, IL-1β upregulated MMP3, MMP13, ADAMTS4, and ADAMTS5 and downregulated collagen II and aggrecan, while these effects were significantly reversed by DSS treatment. All these data demonstrated that DSS efficiently inhibited IL-1β-induced inflammatory responses and ECM degradation in chondrocytes, suggesting that DSS might be a potential therapeutic agent for OA.

Previous studies have identified several signaling pathways involving the action mechanism of IL-1β in OA progression, such as p38 MAPK signaling pathway (Wang 2021), PI3K/Akt/mTOR signaling pathway (Xu 2021), nuclear factor erythroid 2-related factor-2/heme oxygenase-1 signaling pathway (Zhu 2021), JAK2/STAT3 signaling pathway (Liu et al. 2020), and Wnt/β-catenin signaling pathway (Miyatake and Kumagai 2020). Among them, NF-κB signaling is a widely studied pathway participating in IL-1β-induced inflammatory response during OA development (Jimi et al. 2019; Choi et al. 2019). Based on the effects of DSS on inflammatory responses, we focused on the NF-κB signaling pathway. Our results indicated that DSS significantly inhibited p65 phosphorylation and nuclear translocation and promoted IκBα level in the cytoplasm, suggesting a suppressive effect of DSS on activation of NF-κB signaling pathway in chondrocytes. However, whether one or more signaling pathways mediate the effects of DSS during OA progression should be further determined.

DMM model is a widely used in vivo model for studies on the pathogenic processes during OA progression (Glasson 2007). To determine the potential protective role of DSS in OA, an in vivo mouse model was constructed. As expected, DSS efficiently attenuated OA progression in the mouse model. To better define the role of NF-κB signaling in the functions of DSS in OA, in the future, we will apply specific NF-κB signaling inhibitors to further understand the specific mechanism of DSS in OA progression.

## Conclusion

In summary, our results demonstrated that DSS reduced inflammatory responses in OA and efficiently attenuated OA progression in a mouse model, possibly via deactivating the NF-κB signaling pathway, which provided a potential treatment strategy for OA.

## Data Availability

The analyzed data sets generated during the present study are available from the corresponding author on reasonable request.

## Abbreviations

DSS: Danshensu
OA: Osteoarthritis
DMM: Destabilization of the medial meniscus
ECM: Extracellular matrix
IL-1β: Interleukin-1β
IL-6: Interleukin-6
COX-2: Cyclooxygenase 2
TNF-α: Tumor necrosis factor-α
iNOS: Inducible nitric oxide synthase
MMP3: Matrix metalloproteinase 3
MMP13: Matrix metalloproteinase 13
ADAMTS4: A disintegrin and metalloproteinase with thrombospondin motifs 4
ADAMTS5: A disintegrin and metalloproteinase with thrombospondin motifs 5
NF-κB: Nuclear factor-kappa B
